# Activation of the Nrf2/HO-1 pathway by curcumin inhibits oxidative stress in human nasal fibroblasts exposed to urban particulate matter

**DOI:** 10.1186/s12906-020-02886-8

**Published:** 2020-03-30

**Authors:** Ji-Sun Kim, Jeong-Min Oh, Hyunsu Choi, Sung Won Kim, Soo Whan Kim, Byung Guk Kim, Jin Hee Cho, Joohyung Lee, Dong Chang Lee

**Affiliations:** 1 Department of Otorhinolaryngology-Head and Neck Surgery, College of Medicine, The Catholic University of Korea, Seoul, Republic of Korea; 2 Department of Otorhinolaryngology-Head and Neck Surgery, Eunpyeong St. Mary’s Hospital, Seoul, Republic of Korea; 3 Clinical Research Institute, Daejeon St. Mary’s Hospital, Daejeon, Republic of Korea; 4 Department of Otorhinolaryngology-Head and Neck Surgery, Seoul St. Mary’s Hospital, Seoul, Republic of Korea; 5 Department of Otorhinolaryngology-Head and Neck Surgery, Yeouido St. Mary’s Hospital, Seoul, Republic of Korea; 6 Department of Otorhinolaryngology-Head and Neck Surgery, Daejeon St. Mary’s Hospital, Daejeon, Republic of Korea

**Keywords:** Curcumin, ERK, Fibroblast, HO-1, Nrf2, Particulate matter, ROS

## Abstract

**Background:**

Particulate matter (PM) can cause various negative acute and chronic diseases of the respiratory system, including the upper airways. Curcumin has been reported to have anti-inflammatory and anti-oxidative effects; therefore, we investigated the effects of curcumin on nasal fibroblasts exposed to urban PM (UPM).

**Methods:**

Samples of inferior turbinate tissue were obtained from six patients. Flow cytometry was used to assess the levels of reactive oxygen species (ROS) following the treatment of nasal fibroblasts with UPM and/or curcumin. We evaluated the effects of UPM and/or curcumin on the expression of phosphorylated ERK, Nrf2, HO-1, and SOD2 in fibroblasts by Western blotting.

**Results:**

When UPM was applied to nasal fibroblasts, ROS production was significantly increased in a dose-dependent manner. UPM-exposed fibroblasts caused the activation of ERK to increase HO-1 expression and decrease SOD2 expression. Treatment with curcumin reduced the UPM-mediated increase in ROS; this decrease in ROS occurred in a dose-dependent manner. The UPM-induced activation of ERK was inhibited by curcumin. Nrf2 production was also promoted to increase the expression of HO-1 and SOD2 by curcumin.

**Conclusion:**

Curcumin reduced ROS production caused by UPM in human nasal fibroblasts in a dose-dependent manner, suggesting that curcumin has anti-oxidative effects and may be useful in the treatment of nasal diseases caused by UPM, such as allergic and chronic rhinitis.

## Background

Air pollution is a significant environmental risk for humans and is the cause of various diseases and death [1–3]. Particulate matter (PM), which contains organic compounds and harmful metals, is a major source of air pollution. The harmful effects of PM on overall health have been well documented [4–6]. In particular, PM levels have been reported to have various negative acute and chronic effects on the respiratory system, including the upper airways [7–9]. As one of the mechanisms by which PM causes disease in the human body, the generation of oxidative stress following PM exposure has been observed in vitro and in vivo [10, 11]. Therefore, antioxidants that can remove reactive oxygen species (ROS) may help protect against diseases caused by PM.

Fibroblasts modify the structure of the nasal mucosa by controlling production of the extracellular matrix [12]. Fibroblasts also play a key role in chronic inflammation of the nasal cavity by releasing various inflammatory cytokines [13, 14]. We confirmed in previous studies that urban PM (UPM) induces the expression of proinflammatory cytokines in human nasal fibroblasts [15, 16]. These results indicated that upper respiratory diseases such as allergy and chronic rhinitis might be caused by UPM-exposed fibroblasts.

Curcumin (diferuloyl methane) is a yellowish substance present in turmeric isolated from the plant *Curcuma longa*. The therapeutic effects of curcumin have been shown in several studies. Curcumin exhibits anti-inflammatory activity via inhibition of cell signaling pathways by the modulation of several molecular targets; it also exhibits anti-oxidative and anticancer activities by regulating the expression of various enzymes, receptors, and anti-apoptotic proteins [17–19]. While the effects of curcumin in lower respiratory diseases such as asthma, chronic obstructive pulmonary disease (COPD), and pulmonary fibrosis have been extensively investigated [20–22], the impact on upper respiratory diseases, including the nasal cavity, has been poorly studied. In this study, we investigated the effect of curcumin on cultured human nasal fibroblasts exposed to UPM. We evaluated the therapeutic potential of curcumin for UPM-related diseases of the upper airway and assessed the signaling pathways involved.

## Methods

### Reagents

UPM standard reference material (SRM 1648a) was obtained from the National Institutes of Standards and Technology (Gaithersburg, MD, USA). Curcumin, collagenase A, and 5- (and 6-)carboxy-2′,7′-dichlorodihydro-fluorescein diacetate (H2DCFDA) were obtained from Sigma-Aldrich (St. Louis, MO, USA). Antibodies against nuclear factor (erythroid-derived 2)-like 2 (Nrf2), superoxide dismutase (SOD)2, and heme oxygenase (HO)-1 were obtained from Santa Cruz Biotechnology (Santa Cruz, CA, USA). Antibodies against extracellular signal-regulated kinase (ERK), phosphorylated ERK, lamin B1, and glyceraldehyde-3-phosphate dehydrogenase (GAPDH) were obtained from Cell Signaling Technology (Danvers, MA, USA). A stock solution of curcumin was diluted with cell culture medium to confirm the final dose for cell treatment.

### Inferior turbinate tissues

Inferior turbinate tissue was obtained from six patients with turbinate hypertrophy (four males and two females, mean 45.0 ± 14.4 years) who underwent partial inferior turbinectomies to treat nasal obstructions. None of the subjects had a history of asthma or allergy. In addition, the subjects were not treated with antihistamines, oral antibiotics, or intranasal or oral steroids for at least 2 months before tissue harvest. This study, which was approved by our institutional review board, was conducted in accordance with the principles of the Declaration of Helsinki; written informed consent was obtained from the patients prior to surgery.

### Nasal fibroblast cultures

Once removed, the tissues were placed immediately in ice-cold minimal essential medium (MEM)-α, washed three times with antibiotic-antimycotic solution (Gibco, Gaithersburg, MD, USA), washed twice with phosphate-buffered saline (PBS), and cut into small pieces with a sterile scalpel. The tissue samples were incubated in collagenase A (2.5 mg/mL; Sigma-Aldrich) for 90 min at 37 °C and mechanically dissociated. Enzyme activity was stopped by the addition of 0.5 mM ethylenediaminetetraacetic acid. The washed tissue samples were incubated at 37 °C in an atmosphere of 5% CO_2_ in MEM-α containing 10% fetal bovine serum (FBS; Gibco BRL, Grand Island, NY, USA). The nasal fibroblasts were cultured with 10% FBS and 1% penicillin streptomycin solution (Gibco BRL) in MEM-α at 37 °C and 5% CO_2_. The medium was changed every 3 days and all cells used in the experiments were obtained from the fifth cell passage.

### Cell viability assay

To assess the cytotoxicities of UPM and curcumin, nasal fibroblast viability was determined using a MTT Assay Kit (DoGen, Seoul, Republic of Korea). The MTT assay is based on the ability of functional mitochondria to catalyze the reduction of 3-(4,5-dimethylthiazol)-2,5-diphenyltetrazolium bromide to insoluble purple formazan; the concentration of formazan can be measured with a spectrophotometer. Nasal fibroblasts were first cultured in 96-well plates (5 × 10^3^ cells/well) for 24 h, washed twice with PBS, and treated with various concentrations of UPM (0–200 μg/mL) and curcumin (0–5 μM) simultaneously. After 24 h of incubation, the MTT reagent (10 μL) was added and the cells were incubated for an additional 2 h. The absorbance of each cell was measured at 450 ± 20 nm using an ELISA reader (Bio-Rad Laboratories, Hercules, CA, USA) and the percent viability was calculated. The results were reported in units of optical density (OD) and the relative cell viability was calculated using the formula Relative cell viability (%) = (OD treatment / OD control) × 100.

### Quantification of intracellular ROS

Nasal fibroblasts were first cultured in 6-well plates (1 × 10^5^ cells/well) for 24 h, washed twice with PBS, and treated with various concentrations of UPM (0–200 μg/mL) and curcumin (0–5 μM) simultaneously. Cells were harvested for intracellular ROS detection using H2DCFDA. Briefly, the cells were washed twice with PBS and resuspended in Hanks’ Balanced Salt Solution (HBSS) containing 10 μM H2DCFDA. The cell suspensions were incubated at 37 °C for 15 min in the dark and rinsed three times with HBSS to remove excess H2DCFDA. Finally, the cells were collected and the fluorescence intensity was measured by flow cytometry (FACSCanto II; BD Biosciences, Franklin Lakes, NJ, USA).

### Western blotting

Western blotting was performed as described previously [16]. Nasal fibroblasts were first cultured in 100 mm dishes (5 × 10^5^ cells) for 24 h, and then washed twice with PB, and treated with various concentrations of UPM (0–200 μg/mL) and curcumin (0–5 μM) simultaneously. In brief, total cellular proteins were prepared using lysis buffer (Atto, Tokyo, Japan). The protein concentration was measured using a bicinchoninic acid protein assay kit (Pierce, Rockford, IL, USA) and 20 *μ*g amounts, separated by electrophoresis on a 10% sodium dodecyl sulfate-polyacrylamide gel, were then transferred to nitrocellulose membranes (Pall Corporation, Pensacola, Mexico). The membranes were blocked with skimmed milk (5%) in TBS buffer (20 mM Tris, 137 mM NaCl) for 1 h and washed with TBS-T buffer (20 mM Tris, 137 mM NaCl, and 0.05% Tween-20) three times, for 10 min each time. Next, the membranes were incubated with appropriate primary antibodies (1:1000 dilution) overnight at 4 °C. After washing three times with TBS-T, the membranes were incubated with appropriate horseradish peroxidase–conjugated secondary antibodies (1:2000 dilution) for 1 h at room temperature. Proteins were detected using an enhanced chemiluminescence kit (GE, MA, USA) and visualized with the aid of a ChemiDoc XRS+ image analyzer (Bio-Rad, Hercules, CA). Band intensities were derived using ImageLab (Bio-Rad) software.

### Statistical analyses

All data are presented as means ± standard errors of the means (SEMs). All statistical analyses were performed with the aid of GraphPad Prism version 5 software (Graph-Pad, San Diego, CA, USA). We performed one-way analysis of variance (ANOVA) followed by Student’s *t*-test for *P*-value evaluation between two groups or Tukey’s post-hoc test for multiple comparison. *P-*values < 0.05 was considered significant.

## Result

### Effects of curcumin and UPM on nasal fibroblast viability

**Fig. 1:**
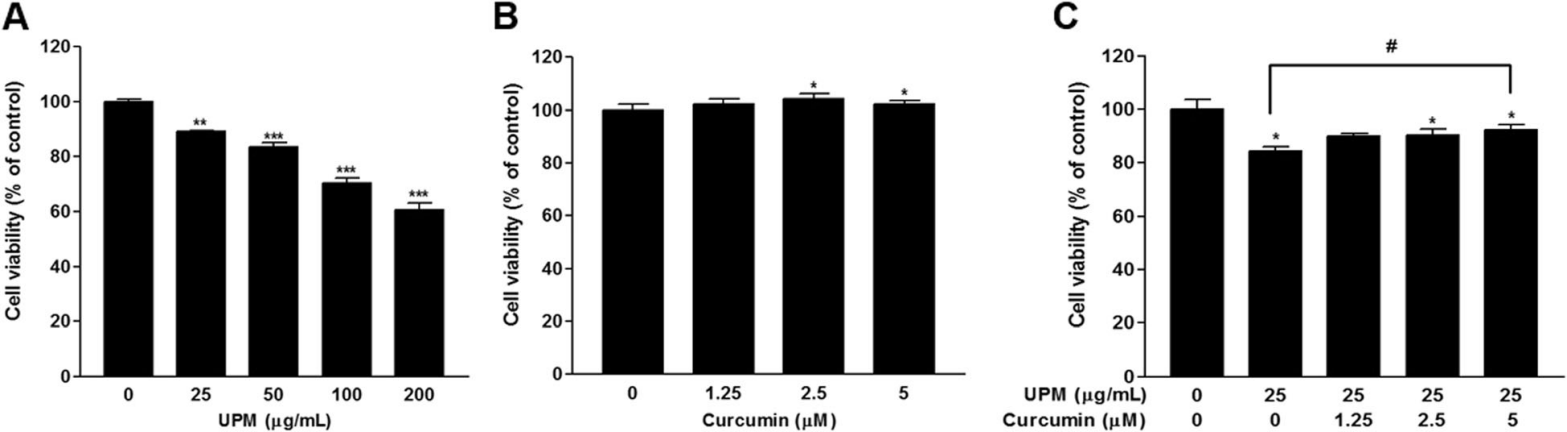
**a** Cell viability as measured by MTT assays of human nasal fibroblasts treated with various concentrations of UPM for 24 h. **b** Cell viability as measured by MTT assays of nasal fibroblasts treated with various concentrations of curcumin for 24 h. **c** Cell viability as measured by MTT assays of nasal fibroblasts treated with various concentrations of curcumin and 25 μg/mL of UPM for 24 h. Each value in the graph represents the mean ± SEM of three independent experiments. *p < 0.05, **p < 0.01, and ***p < 0.001 vs. 0 μg/mL of UPM or 0 μM of curcumin; #*p* < 0.05 vs. 25 μg/mL of UPM. MTT: 3-(4,5-dimethylthiazol-2-yl)-2,5-diphenyltetrazolium bromide; UPM: urban particulate matter; SEM: standard error of the mean

Nasal fibroblasts were cultured with curcumin and/or UPM at varying concentrations for 24 h, and the effect on viability was determined by MTT assays. Treatment with UPM (> 25 μg/mL) decreased the viability of nasal fibroblasts significantly in a dose-dependent manner (Fig. 1a), while treatment with curcumin (> 2.5 μM) increased its viability significantly (*p* < 0.05) (Fig. 1b). The reduction in viability caused by UPM (25 μg/mL) was reversed by curcumin, and this effect was significant at 5 μM curcumin (*p* < 0.05) (Fig. 1c).

### Effects of UPM on ROS generation in nasal fibroblasts

**Fig. 2:**
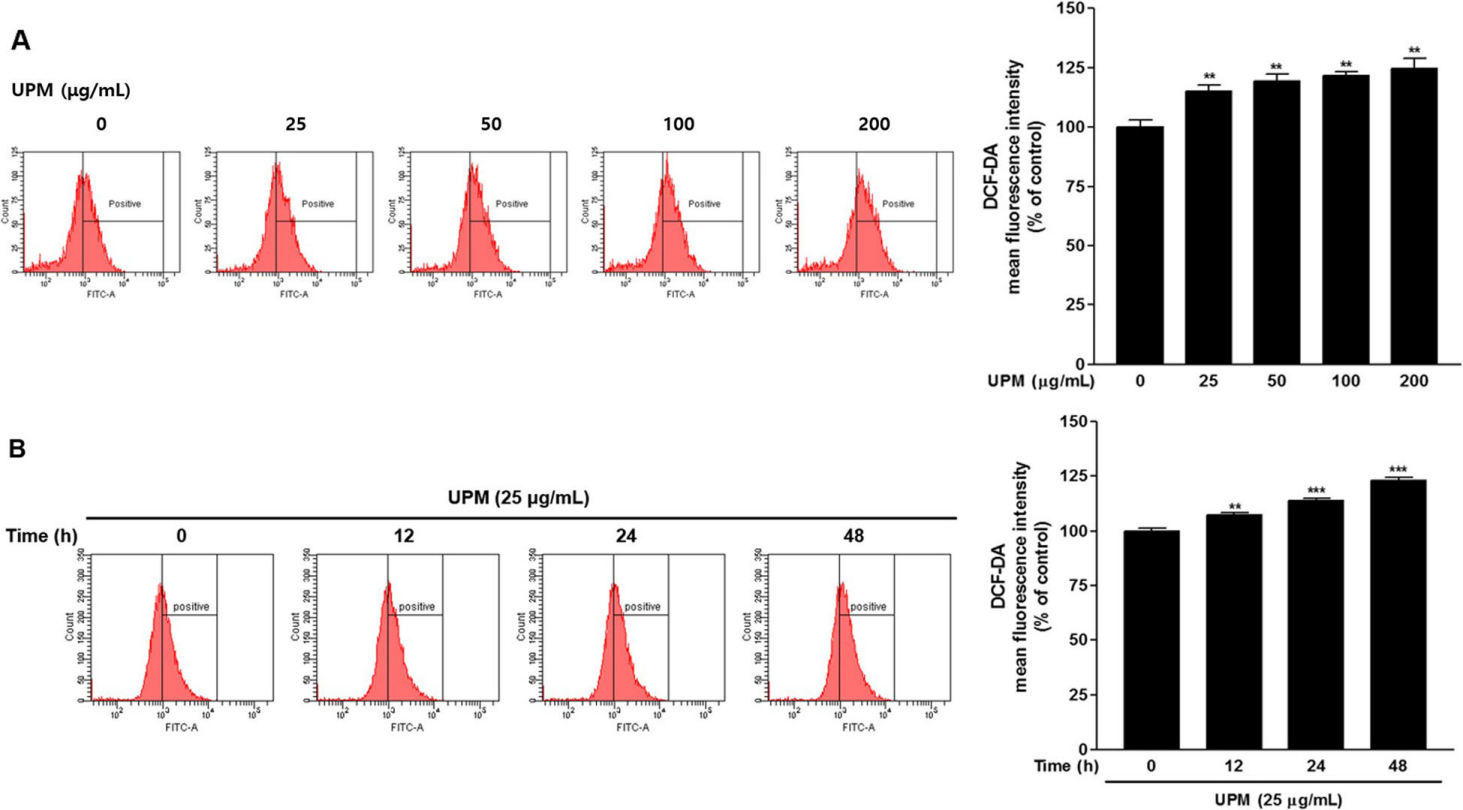
ROS generation in human nasal fibroblasts treated with UPM as measured by flow cytometry. **a** Dose-dependent ROS generation in human nasal fibroblasts exposed to UPM. **b** Time-dependent ROS generation in human nasal fibroblasts exposed to UPM. ROS were quantified by flow cytometry with DCF-DA. Data are presented as the mean ± SEM. *p < 0.05, **p < 0.01, and ***p < 0.001 vs. the control group. ROS: reactive oxygen species; UPM: urban particulate matter; DCF-DA: 2′,7′-dichlorofluorescin diacetate; SEM: standard error of the mean

Nasal fibroblasts were stained with DCF-DA to determine ROS levels by flow cytometry. When UPM was applied to nasal fibroblasts at concentrations > 25 μg/mL, ROS production was significantly increased in a dose-dependent manner compared with the controls (*p* < 0.01) (Fig. 2a). ROS production in fibroblasts increased significantly after 12 h of exposure to UPM (25 μg/mL) compared with the controls (Fig. 2b).

### Effects of UPM on the phosphorylation of ERK and expression of HO-1 and SOD2 in nasal fibroblasts

**Fig. 3:**
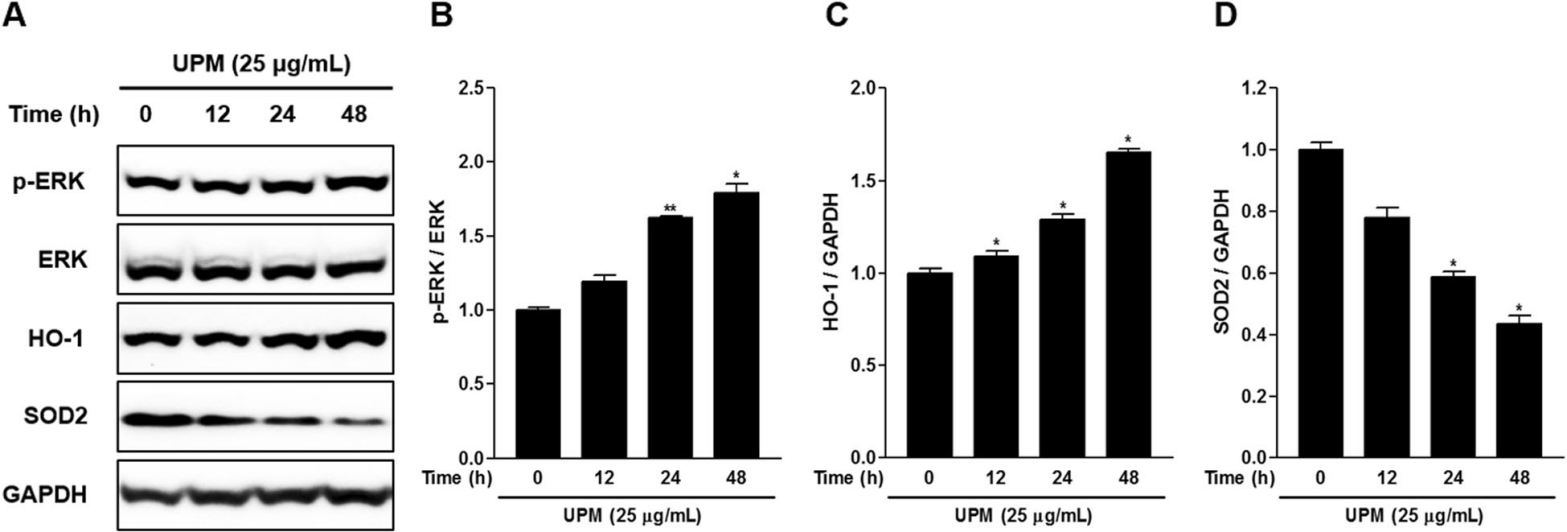
Expression of HO-1 and SOD2 and phosphorylation of ERK following the exposure of nasal fibroblasts to UPM. **a** Western blot analysis of phosphorylated ERK (p-ERK), total ERK, HO-1, and SOD2 in human nasal fibroblasts treated with UPM (25 μg/mL) for the indicated durations. **b** Density ratio of p-ERK to ERK. **c** Density ratio of HO-1 to GAPDH. **d** Density ratio of SOD2 to GAPDH. Data are presented as the mean ± SEM. *p < 0.05 and **p < 0.01 vs. the control group. ERK: extracellular signal-regulated kinase; HO-1: heme oxygenase-1; SOD2: superoxide dismutase 2; UPM: urban particulate matter; GAPDH: glyceraldehyde-3-phosphate dehydrogenase

We evaluated the time dependency of the effect of UPM on the phosphorylation of ERK as well as the expression of HO-1 and SOD2 in nasal fibroblasts by Western blotting. The levels of phosphorylated ERK and expression of HO-1 increased with increasing UPM treatment time compared with the controls (Fig. 3). By contrast, SOD2 expression decreased as nasal fibroblasts were exposed to UPM for longer periods of time compared with the controls (Fig. 3d).

### Effects of curcumin on UPM-induced ROS production in nasal fibroblasts

**Fig. 4:**
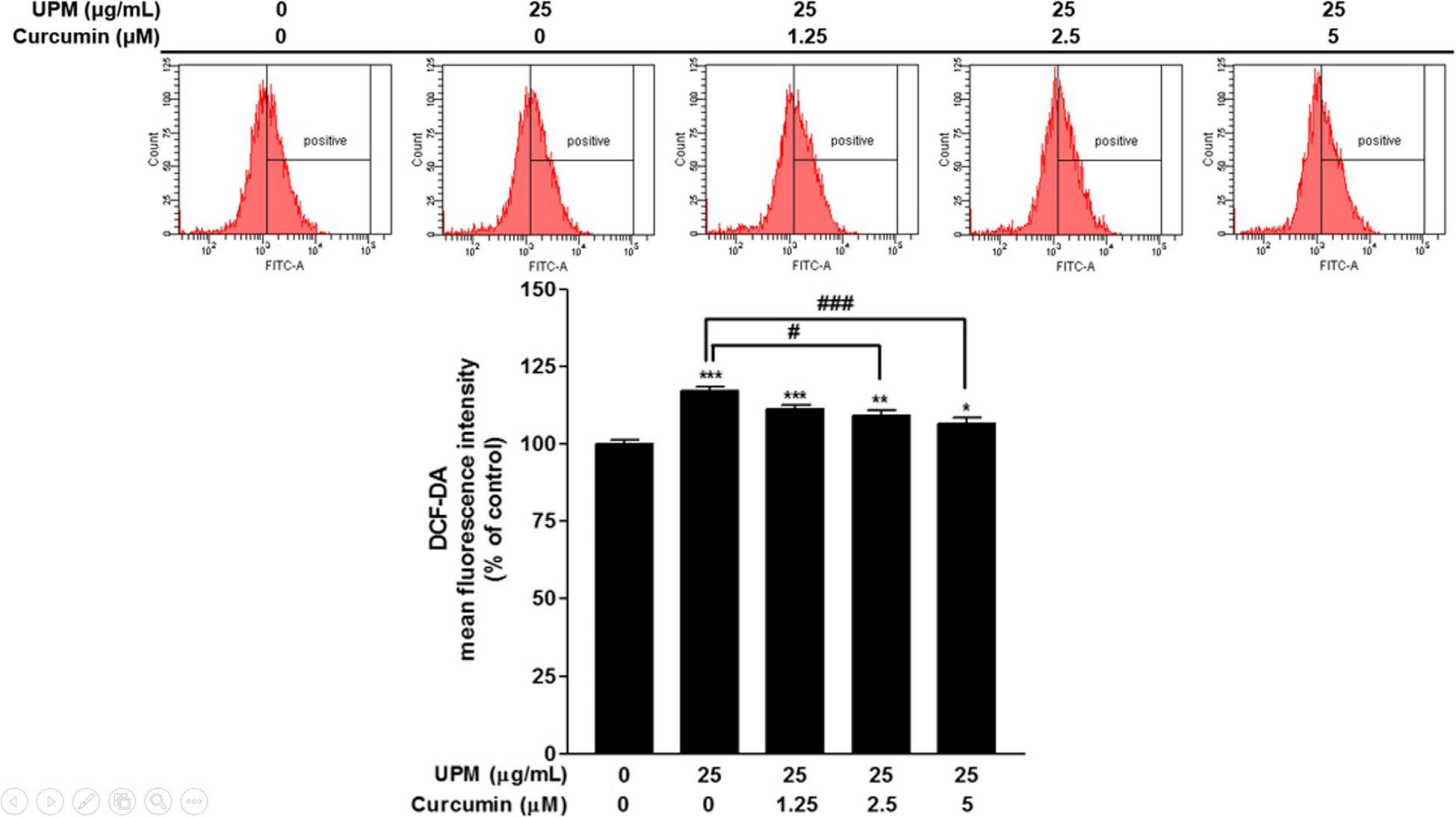
Effects of curcumin on the UPM-induced production of ROS in nasal fibroblasts. ROS were quantified by flow cytometry with DCF-DA. Each value in the graph represents the mean ± SEM of three independent experiments. *p < 0.05, **p < 0.01, and ***p < 0.001 vs. 0 μg/mL of UPM or 0 μM of curcumin; #*p* < 0.05 and ###*p* < 0.001 vs. 25 μg/mL of UPM. UPM: urban particulate matter; ROS: reactive oxygen species; DCF-DA: 2′,7′-dichlorofluorescin diacetate; SEM: standard error of the mean

To evaluate the effect of curcumin on UPM-induced ROS production, flow cytometry with DCF-DA was performed. Nasal fibroblasts were treated with curcumin (0, 1.25, 2.5, or 5 μM) and UPM (25 μg/mL) for 24 h simultaneously. ROS levels were significantly increased following UPM-alone treatment compared with the non-treatment group (*p* < 0.001); however, when curcumin was added, ROS levels decreased in proportion to the curcumin dose (Fig. 4). In particular, treatment with curcumin (2.5 or 5 μM) resulted in a statistically significant reduction in ROS levels in UPM-exposed fibroblasts compared with treatment with UPM alone (Fig. 4).

### Effects of curcumin on ERK and Nrf2, and their downstream targets SOD2 and HO-1, in UPM-exposed nasal fibroblasts

**Fig. 5:**
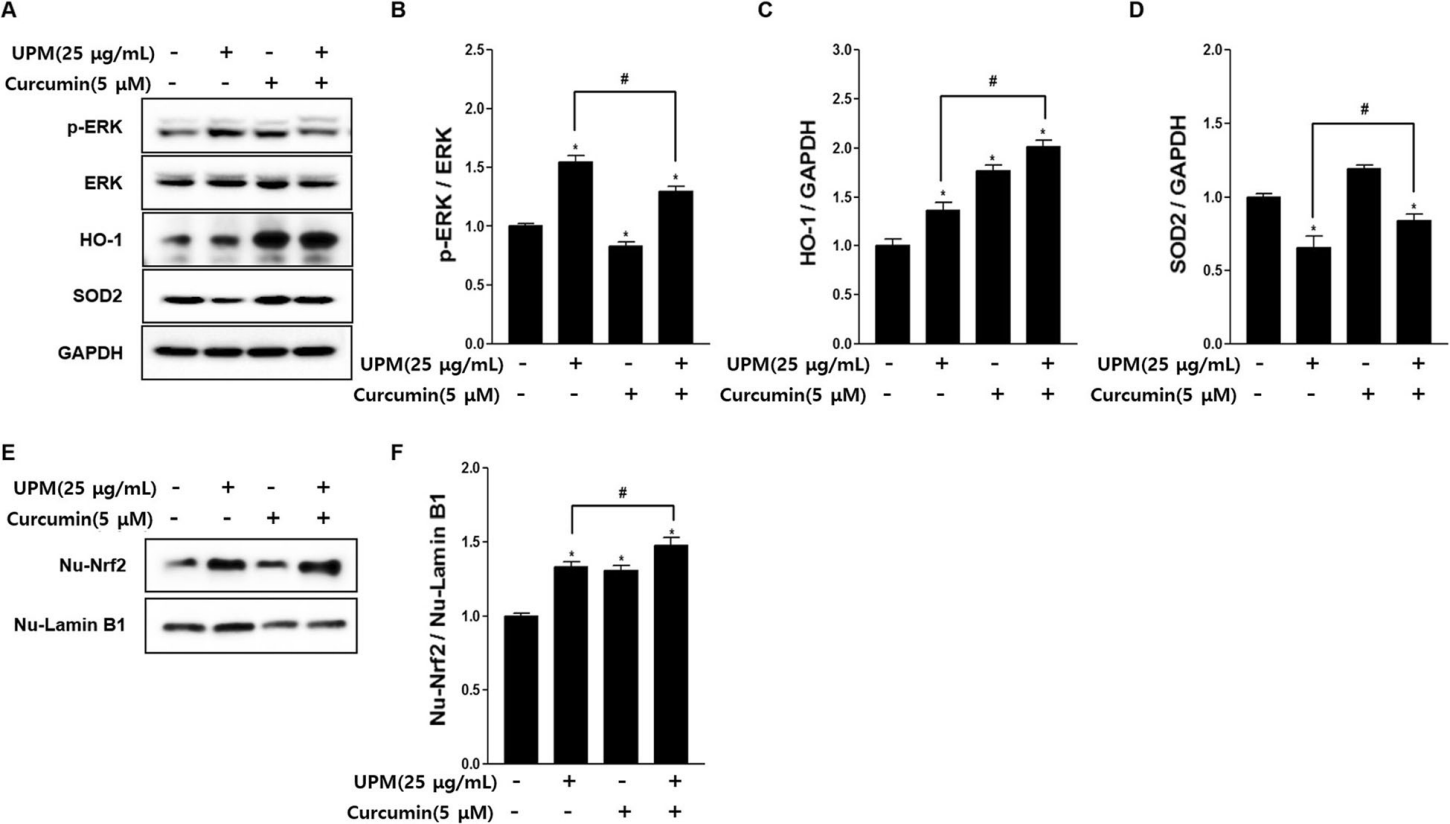
**a** Representative Western blots of ERK, SOD2, and HO-1 in fibroblasts treated with 5 μM of curcumin and 25 μg/mL of UPM for 24 h. **b** Density ratio of p-ERK to ERK. **c** Density ratios of HO-1 to GAPDH. **d** Density ratios of SOD2 to GAPDH. **e** Representative Western blots of Nrf2 in fibroblasts treated with 5 μM of curcumin and 25 μg/mL of UPM for 4 h. **f** Density ratio of nuclear Nrf2 to nuclear lamin B1. Each value of the graph represents the mean ± SEM of three independent experiments. *p < 0.05 vs. 0 μg/mL of UPM and 0 μM of curcumin; #*p* < 0.05 vs. 25 μg/mL of UPM. ERK: extracellular signal-regulated kinase; HO-1: haem oxygenase-1; SOD2: superoxide dismutase 2; UPM: urban particulate matter; Nrf2: nuclear factor (erythroid-derived 2)-like 2

To assess the role of ERK and Nrf2 in the effects of curcumin on nasal fibroblasts, we assayed phosphorylated ERK, nuclear Nrf2, SOD2, and HO-1 expression by Western blotting following treatment with curcumin (5 μmol/L) and UPM (25 μg/mL), alone or in combination. Curcumin significantly reduced the levels of phosphorylated ERK induced by UPM after 24 h of treatment compared with UPM alone (*p* < 0.05) (Fig. 5b). On the other hand, expression of HO-1 and SOD2 was significantly increased following treatment with curcumin compared with UPM alone (*p* < 0.05) (Fig. 5c). Following treatment with curcumin and UPM for 4 h, nuclear Nrf2 was significantly increased compared with treatment with UPM alone (*p* < 0.05) (Fig. 5e).

## Discussion

In this study, we determined that treatment with UPM induced ROS production, which activated ERK to increase HO-1 expression and decrease SOD2 expression. Curcumin reduced the UPM-mediated increase in ROS levels in a dose-dependent manner. Our results suggest that curcumin decreased the activation of ERK mediated by UPM, and increased Nrf2 directly to increase expression of HO-1 and SOD2.

PM is classified according to particle size. Particles less than 10 μm are referred to as PM_10_, while particles less than 2.5 μm are called PM_2.5_ [23]. Coarse PM (PM_2.5–10_), which includes all PM_10_ except PM_2.5_, is mainly deposited in the nasal cavity, oral cavity, and upper bronchus. PM_2.5_ is inhaled deeper into the lungs; some of it is deposited in the alveoli, and some of it enters the pulmonary circulation, affecting the entire body [5]. The upper airway, including the nasal cavity and oral cavity, is the first gateway for PM to enter the body. Several studies have been published on the effects of PM on the upper airway; for example, long- and short-term exposure to PM can lead to allergic rhinitis [24, 25]. PM has also been shown to cause non-allergic symptoms in rhinosinusitis patients [26]. In cell experiments, PM exposure was observed to interfere with the barrier function of the epithelial layer by disintegrating the tight junctions of nasal epithelial cells [27].

ROS is a by-product of oxygen metabolism in aerobic organisms and can damage cellular molecules, such as proteins, nucleic acids, and lipids [28]. The cells activate antioxidant mechanisms to protect against the toxicity of ROS [29]. Oxidative stress is a state of imbalance between antioxidant mechanisms and ROS levels [28]. Oxidative stress due to high concentrations of ROS increases the proliferation of certain subsets of adaptive immune cells and increases inflammatory cytokine release [30, 31]. One study reported that PM_2.5_ increased ROS and inflammatory cytokines in nasal epithelial cells, decreasing cell viability [32]. This suggested that oxidative stress and inflammatory responses due to PM_2.5_ adversely affect the growth of nasal epithelial cells. In the present study, UPM decreased the viability of human nasal fibroblasts (Fig. 1). Flow cytometry also revealed that ROS production was increased in nasal fibroblasts exposed to UPM. The increased ROS levels were proportional to the concentration and exposure time of UPM (Fig. 2).

HO-1 is an important player in the cellular defense against oxidative stress and is highly induced by inflammatory cytokines [33]. Given that HO-1 is induced by a protective mechanism in response to harmful stimuli, the induction of HO-1 could be considered as a therapeutic approach for protection against oxidative stress [34]. SODs are important cell-defense redox enzymes that convert superoxide radicals into oxygen and hydrogen peroxide. SOD2 is responsible for maintaining ROS homeostasis under inflammatory conditions [35]. Our results demonstrate that inflammatory reactions in UPM-exposed nasal fibroblasts induce HO-1 and reduce SOD2, which maintains ROS homeostasis. The ERK signaling pathway was also activated in this process (Fig. 3).

Curcumin has been reported to exhibit anti-oxidative and anti-inflammatory effects with bioprotective properties [17]. There have been many studies of the therapeutic effects of curcumin in various diseases, including lower respiratory disease. Curcumin has shown potential for the treatment of respiratory diseases such as COPD, asthma, pulmonary fibrosis, and acute lung injury [20–22, 36]. By contrast, few studies have reported the effects of curcumin on upper respiratory disease. In studies of rat models of allergic rhinitis, curcumin has been shown to increase antioxidant enzymes and reduce inflammatory cytokines in tissues and serum [37, 38]. One study of perennial allergic rhinitis patients reported that curcumin relieved sneezing and rhinitis [39].

Nrf2 is a transcription factor responsible for regulating cellular redox balance and phase II detoxification [40, 41]. When cells are exposed to oxidative stress, Nrf2 induces the expression of many protective genes, resulting in increased levels of antioxidants [42]. Under normal or unstressed conditions, Nrf2 is sequestered in the cytoplasm by Keap1. Under conditions of stress, the modification of Keap1 or phosphorylation of Nrf2 promotes the dissociation of Nrf2 from Keap1 [43]. Nrf2 then moves to the nucleus, activating the transcription of genes (e.g., those encoding HO-1 and SOD2) that have detoxifying and anti-oxidative effects [44]. In the present study, curcumin activated Nrf2 while inhibiting ERK in UPM-exposed fibroblasts. This resulted in a significant increase in the antioxidant enzymes HO-1 and SOD2 (Fig. 5).

Although this study was conducted in cell culture, it is the first to investigate the effects of curcumin on upper airway cells exposed to PM.

## Conclusions

The results of this study show that curcumin reduced ROS production caused by UPM in human nasal fibroblasts in a dose-dependent manner, suggesting that curcumin is effective in the treatment of nasal diseases due to UPM exposure.

## Data Availability

The datasets used and/or analyzed during the current study is available from the corresponding author on reasonable request.

## Abbreviations

PM: particulate matter
UPM: urban particulate matter
ROS: reactive oxygen species
COPD: chronic obstructive pulmonary disease
Nrf2: Antibodies against nuclear factor (erythroid-derived 2)-like 2
HO: heme oxygenase
SOD: superoxide dismutase
ERK: extracellular signal-regulated kinase
GAPDH: glyceraldehyde-3-phosphate dehydrogenase
MEM: minimal essential medium
PBS: phosphate-buffered saline
FBS: foetal bovine serum
